# Right Ventricular Lead Implantation Facilitated By A Guiding Sheath In A Patient With Severe Chamber Dilatation With Tricuspid Regurgitation

**Published:** 2011-10-02

**Authors:** Phang Boon Lim, David C Lefroy

**Affiliations:** Department of Cardiac Electrophysiology, Imperial College Healthcare NHS Trust, Cardiology Department, St Mary's Hospital, Pread Street, London W2 1NY

**Keywords:** Restrictive cardiomyopathy, giant atria, guiding sheaths, pacemaker

## Abstract

Implantation of pacemakers can be challenging in the context of dilated cardiac chambers and valvular regurgitation. We report a difficult case of single chamber pacemaker implantation in a patient with restrictrive cardiomyopathy resulting in grossly enlarged atria and severe tricuspid regurgitation. In this situation, use of a slittable guiding sheath, more typically used for coronary sinus lead implantation, greatly facilitated rapid and stable deployment of the right ventricular lead.

## Case Report

**Figure 1:**
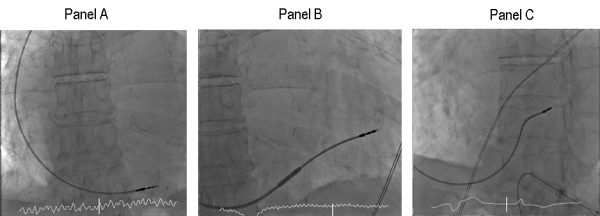
Panel A: AP projection showing final lead position following first procedure, with lead displacement detected the next day. Panel B: AP projection, showing pacing lead supported inside slittable sheath in final position. Panel C: LAO projection, final lead position in the right ventricular septum after sheath slitted and removed.

An 88 year old man presented with syncope following several pre-syncopal episodes over the previous 24 hours. An ECG showed atrial fibrillation with a broad QRS with a ventricular rate of 30bpm. Transthoracic echocardiography showed giant left and right atria (each with an estimated area of 60cm^2^ on the apical 4 chamber view), with structurally normal valves and functional mitral and tricuspid regurgitation, consistent with a diagnosis of restrictive cardiomyopathy.  An initial attempt at single chamber pacing using a screw-in right ventricular lead was difficult due to lead instability and took 3 hours (Fluoroscopy time 46 minutes, dose 6777 cGycm2) (Figure 1, Panel A). Lead displacement was detected the following day. During the repeat procedure, a 45cm 9F, inner lumen 7.2F, multipurpose slittable sheath (Medtronic Attain Command 6250-MB2) was advanced into the right ventricle with support from a 6F multipurpose catheter and 0.035" J-wire. A 7F active fixation 60cm pacing lead (Boston Scientific model no. 4137) was advanced to the right ventricular septum and a stable position was obtained (Figure 1, Panels B and C). The guiding sheath was removed by being slit in the conventional manner. The repeat procedure took 45 minutes (Fluoroscopy time 7 minutes, dose 876 cGycm2). The lead remained stable with satisfactory pacing and radiographic parameters and the patient was discharged the following day.

## Discussion

Guiding sheaths are commonly used to facilitate coronary sinus lead implants [1] and pacing lead implants in paediatric patients [2]. However, these sheaths tend not to be used for right ventricular lead implants in the general adult population. In this case, the support and extra reach provided by a guiding sheath allowed firm and stable contact between the pacing lead tip and the endocardium during distal screw deployment despite the presence of massive chamber dilatation and severe tricuspid regurgitation.

Other strategies that could be considered in difficult pacing cases are alternative lead placement strategies by using either pre-shaped stylets (originally distributed by St Jude Medical to facilitate septal lead placement) or self-shaped stylets. If apical positioning is difficult, then placement of the lead into the right ventricular outflow tract septum can be facilitated by shaping a stylet with a generous distal curve with a swan neck deformity (angulated posteriorly). The whole lead, with the stylet either fully inserted, or pulled back from the tip, is then inserted into the pulmonary artery initially, before being pulled down into a stable right ventricular outflow tract septal position [3].  The use of broad curvatures on the stylets with steroid-eluting active fixation leads can facilitate implants in patients with severe tricuspid regurgitation following annuloplasty ring repair [4].

Other strategies that might be considered include left ventricular lead placement through the coronary sinus [5], or use of epicardial pacing systems [6]. These techniques may be suitable in patients who have prosthetic tricuspid valves.

During acute implantation, the presence of a stable R wave suggests a good pacing site. Recent studies have shown that the current of injury is better than R wave, slew rate and impedance measurements at predicting acute stability during active fixation lead implants [7,8]. In challenging implants, it is worthwhile optimising these lead parameters before accepting the final lead position.

Alternative lead placement strategies, including the use of appropriately-selected tools may greatly facilitate difficult cases, even when these tools are not part of conventional equipment typically used for single chamber pacemaker implantation.

## References

[R1] Abraham WT (2002). Cardiac resynchronization in chronic heart failure. The New England Journal of Medicine.

[R2] Cantu F (2009). Selective-site pacing in paediatric patients: a new application of the Select Secure system. Europace.

[R3] Mond HG (2007). The right ventricular outflow tract: the road to septal pacing. Pacing Clin Electrophysiol.

[R4] Kistler PM (2002). The challenge of endocardial right ventricular pacing in patients with a tricuspid annuloplasty ring and severe tricuspid regurgitation. Pacing Clin Electrophysiol.

[R5] Lee ME (1983). Special considerations in ventricular pacing in patients with tricuspid valve disease. The Annals of thoracic surgery.

[R6] Cooper JP (1995). Permanent pacing in patients with tricuspid valve replacements. British heart journal.

[R7] Redfearn DP (2007). Current of injury predicts acute performance of catheter-delivered active fixation pacing leads. Pacing Clin Electrophysiol.

[R8] Saxonhouse SJ (2005). Current of injury predicts adequate active lead fixation in permanent pacemaker/defibrillation leads. J Am Coll Cardiol.

